# Case report: a case of diffuse unilateral subacute neuroretinitis (DUSN) in a child

**DOI:** 10.1186/s12886-018-0854-7

**Published:** 2018-09-14

**Authors:** David S Curragh, Anne Ramsey, Sharon Christie, Eibhlin McLoone

**Affiliations:** 1 Department of Ophthalmology, Royal Victoria Hospital, 274 Grosvenor Road, Belfast, BT12 6BA Northern Ireland; 2 Royal Hospital for Sick Children, Royal Victoria Hospital, Belfast, Northern Ireland

**Keywords:** Diffuse, Unilateral, Subacute, Neuroretinitis, Paediatric, Uveitis

## Abstract

**Background:**

Diffuse unilateral subacute neuroretinitis (DUSN) is a rare cause of posterior uveitis in the United Kingdom. It typically presents unilaterally in children and young adults but rarely bilateral cases have been reported. It is also rare to have multiple worms in the same eye causing the clinical picture. In this article, we present a challenging case of DUSN in a young girl unresponsive to conventional treatments suggesting the possibility of multiple worms being present in the same eye.

**Case Presentation:**

An 8-year-old girl presented with a 2-month history of headaches. On occasions the headaches were associated with redness and watering of her left eye. She denied any visual loss or visual symptoms. Her visual acuity was reduced to 6/30 in her left eye. Fundal examination revealed a unilateral chorioretinitis. Investigation did not reveal a specific cause for the chorioretinitis. Over 15 months her visual acuity improved to 6/9 but the fundal appearance changed and a diagnosis of DUSN was made. She was treated with focal laser, systemic anti-helminthic and immunosuppressive treatments but continued to develop new, active areas of chorioretinitis, raising the possibility of multiple worms in the sub-retinal space. There is also a concern as to other central nervous system (CNS) involvement given her significant and ongoing headaches.

**Conclusion:**

We present a challenging case of DUSN in a young girl; a condition that remains rare in the UK. She was unresponsive to both focal laser and systemic anti-helminthic and immunosuppressive treatments suggesting the possibility of multiple worms being present in the sub-retinal space. This case highlights the difficulties often encountered in the treatment of DUSN, even when a worm can be identified. Her visual prognosis is poor as there was ongoing recurrence of active chorioretinitis.

## Background

Diffuse unilateral subacute neuroretinitis (DUSN) is a rare cause of posterior uveitis in the United Kingdom [1]. The cause of inflammation remained unknown until association with a nematode was proposed in 1983 [2, 3]. It typically presents unilaterally in children and young adults but rarely bilateral cases have been reported [4]. Entry of the worm into the eye occurs when nematode eggs, after being ingested, find their way into the eye haematogenously. For months to years the worm can wander in the sub-retinal space [2]. Clinical features of DUSN can be divided into early and late. Early features include mild-to-moderate vitritis, papillitis, retinal vasculitis, grey–white or yellow–white evanescent lesions located in the deep retinal layers and retinal pigmentary epithelial (RPE) changes [5]. Late features include multifocal choroiditis episodes, increase in the internal limiting membrane reflex (Oréfice’s sign), diffuse retinal atrophy, narrowing of retinal vessels and optic disc atrophy [5]. Treatment of DUSN can be difficult. Laser photocoagulation remains the most successful treatment when the worm can be visualised, but visualisation is only reported in 25–40% of patients [6, 7]. Treatment with systemic anti-helmenthic therapy is an option that has been used with variable success and immunosuppressive therapy has been proposed. It is also rare to have multiple worms in the same eye causing the clinical picture. In this article, we present a challenging case of DUSN in a young girl unresponsive to conventional treatments suggesting the possibility of multiple worms being present in the same eye.

## Case Presentation

### History

An 8-year-old girl presented in August 2015 to the Royal Belfast Hospital for Sick Children’s Accident and Emergency department with headache for 2 months. These had been increasing in intensity and frequency for the preceding 3 weeks requiring her to take paracetamol on a daily basis. At times the headache was associated with redness and watering of her left eye. She attended her optician due to the headaches who noted an abnormal appearance of her left fundus. She denied any visual loss or any other visual symptoms. There were no previous eye problems known.

Systemic enquiry revealed she had occasional nosebleeds. There was no foreign travel except for a family holiday in Spain twelve months previously. As her grandfather was from South Africa, she had received the Bacillus Calmette-Guérin (BCG) vaccine. Her family had a pet dog that lives outside. There were no cats and she did not live on a farm. There was no history of tick bites or cat scratches. She reported no recent viral illness or vaccinations and no recent pyrexia, fatigue, cough or sore throat. She had no known underlying medical conditions. She was not on any medications except for paracetamol as required for her headaches.

Aged 7, she had previously been admitted for investigation of pyrexia and left knee swelling. At that time, mum described her as having a bruise-like rash around the affected left knee. She was extensively investigated as a Magnetic Resonance Imaging (MRI) scan showed abnormal changes in and around both knees. Bilateral bone marrow trephine of each posterior iliac crest and bone morrow aspirate and biopsy of the affected distal femur were all negative. Haematological malignancy was excluded. Of note, at that time her C-Reactive Protein (CRP) was 69 mg/L and Erythrocyte Sedimentation Rate (ESR) was 110 mm/hr. but otherwise blood testing did not identify any specific underlying diagnosis for her peculiar presentation.

### Examination findings

**Fig. 1:**
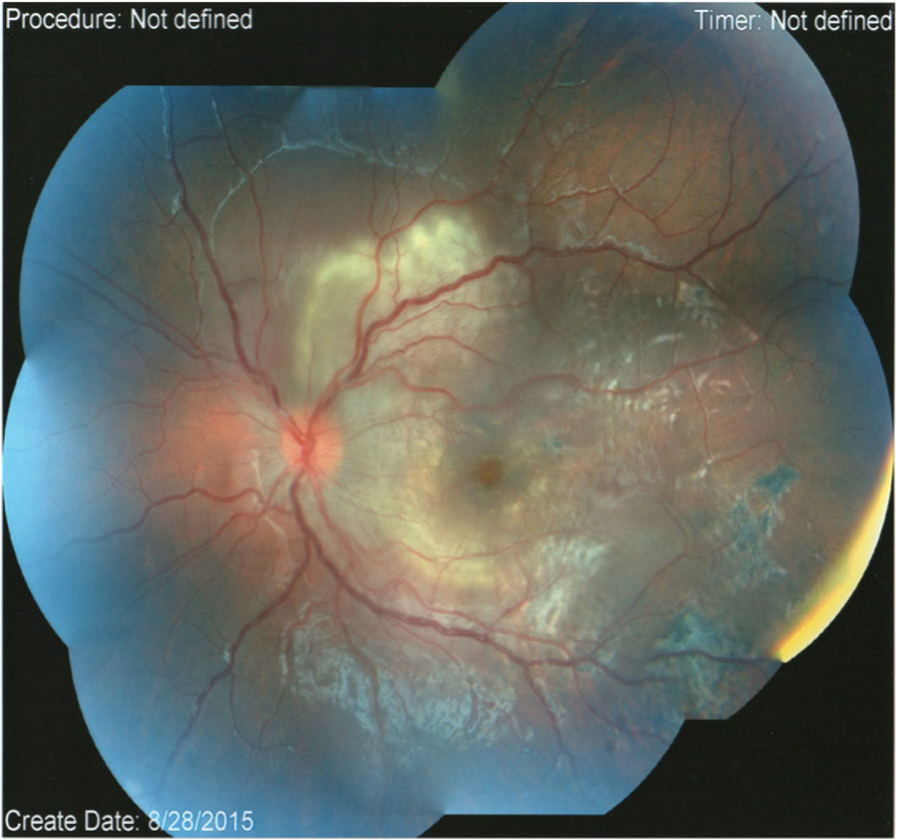
Colour fundal photograph of the left eye at presentation showing white sub-retinal infiltrate involving the nasal macula and retina superior to the disc. There was mild vessel tortuosity, disc swelling, temporal chorioretinal pigmentation and increase in the internal limiting membrane reflex (Oréfice’s sign)

The examination findings at presentation are shown in Table 1 and Fig. 1.

**Table 1:** Examination findings at presentation

RIGHT		LEFT
6/6	Visual Acuity	6/30
Quiet	Anterior Segment	Quiet
Clear	Lens	Clear
Quiet	Vitreous	Cells+
Normal	Disc	Hyperaemic
Normal	Macula	White sub-retinal infiltrate extending superior to macula
Normal	Vessels	Mild tortuosity
Normal	Peripheral retina	Temporal chorioretinal pigmentation

### Management

She was admitted with findings of left posterior uveitis and mild vitritis for further investigation. A differential diagnosis of unilateral chorioretinitis included infectious chorioretinitis such as toxoplasma, toxocara, tuberculosis, syphilis, borriella burgdorferi (*B.burgdorferi*) and post-streptococcal syndrome and inflammatory chorioretinitis such as sarcoidosis, multifocal choroiditis and other white dot syndromes. An infiltrative cause was also considered.

### Investigations

Extensive investigations, as presented in Table 2, failed to identify a specific cause for her unilateral chorioretinitis.

**Table 2:** Investigations

Serology	
Full blood count	Normal
Urea and electrolytes	Normal
Liver function test	Normal
Erythrocyte sedimentation rate (ESR)	Normal
C-Reactive protein (CRP)	Normal
Anti-nuclear antibodies	Positive
Anti-Streptolysin O titre	200 units/ml (Normal)
IgG/IgM Toxoplasma	Negative
IgG/IgM Toxocara	Negative
IgG/IgM *B.burgdorferi*	Negative
IgM Herpes simplex virus	Negative
IgM Cytomegalovirus	Negative
IgM Varicella zoster virus	Negative
IgM Parvovirus B19	Negative
Imaging	
MRI brain	Normal
Magnetic Resonance Venogram brain	Normal
Magnetic Resonance Angiogram brain	Normal
Chest X-ray	Normal
CSF Analysis	
White cell count	< 1 × 10^6^ /L)
Total protein	0.26 g/L
Glucose	2.9 mmol/L
Culture	Negative
Polymerase Chain Reaction (PCR) for herpes simplex virus	Negative
PCR for Enterovirus	Negative
PCR for Parechovirus	Negative
PCR for Streptococcus pneumoniae	Negative
PCR for Haemophilus influenza	Negative
PCR for Cytomegalovirus	Negative
PCR for Bartonella	Negative
PCR for Treponema palladium	Negative
Other	
Mantoux	Positive (but had BCG vaccine)
Quantiferon	Negative
Gastric washings	No acid-fast bacilli

### Clinical progress

**Fig. 2:**
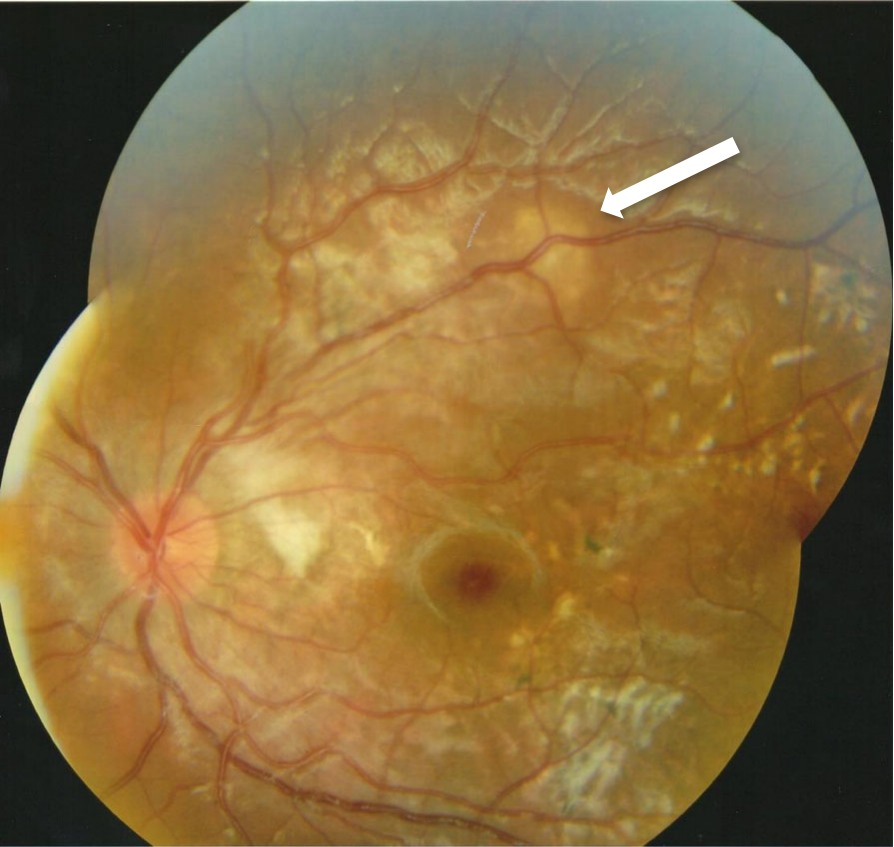
Colour fundal photograph of the left eye 4 months after presentation showing an improved clinical picture with reduced subretinal infiltration but a focal area of chorioretinitis along the superotemporal arcade (white arrow)

**Fig. 3:**
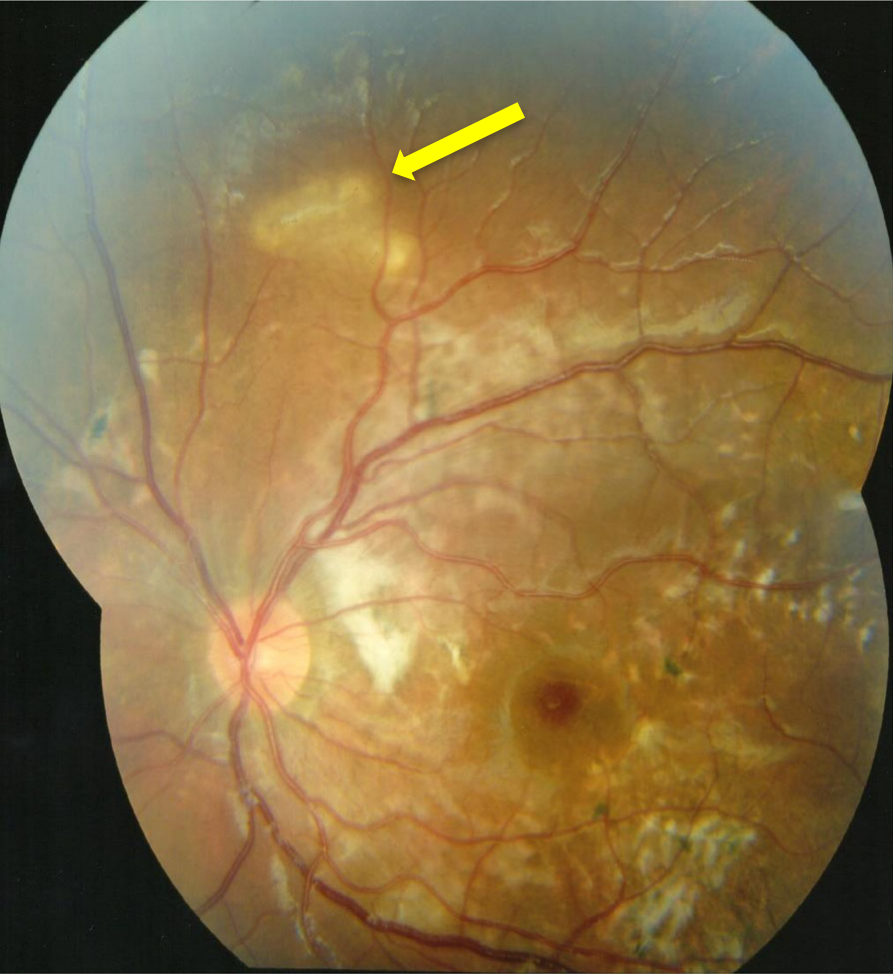
Colour fundal photograph of left eye 5 months following presentation demonstrating mild but reduced optic disc oedema, white subretinal fibrosis temporal to the disc, scattered retinal pigment epithelial proliferation (pigmented scars) and an active area of chorioretinitis superior to the disc with a white glistening nematode as indicated by the yellow arrow

**Fig. 4:**
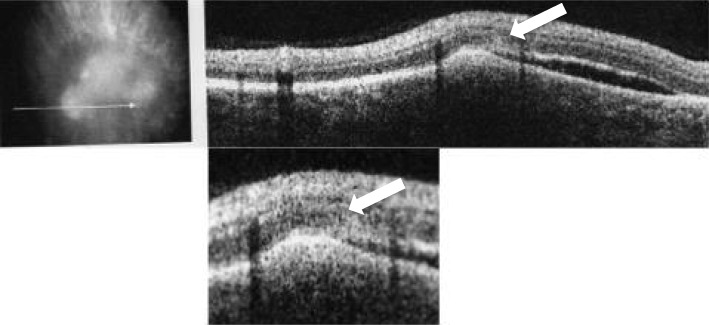
Ocular coherence tomogram horizontally through the area of active chorioretinitis superior to the disc demonstrating a nematode with tapered end curling upwards from the sub-retinal space into deep retinal layers as indicated by the white arrow (standard and magnified views shown)

Over the following 4 months, without treatment, her vision increased in the left eye to 6/12 unaided and the fundal appearance improved showing reduced sub-retinal infiltration; however, a focal circular area of chorioretinitis developed along the superotemporal arcade (Fig. 2). One month later, the circular area of chorioretinitis had faded but a new active area of chorioretinitis was noted superior to the disc. Within this area, an elongated, white, glistening nematode was identified with an estimated size of 1500 μm when compared with the optic disc diameter (Fig. 3). Ocular coherence tomography horizontally through this new area of active chorioretinitis indicated a nematode with tapered end curling upwards from subretinal space into deep retinal layers (Fig. 4). This led to a working diagnosis of diffuse unilateral subacute neuroretinitis (DUSN).

**Fig. 5:**
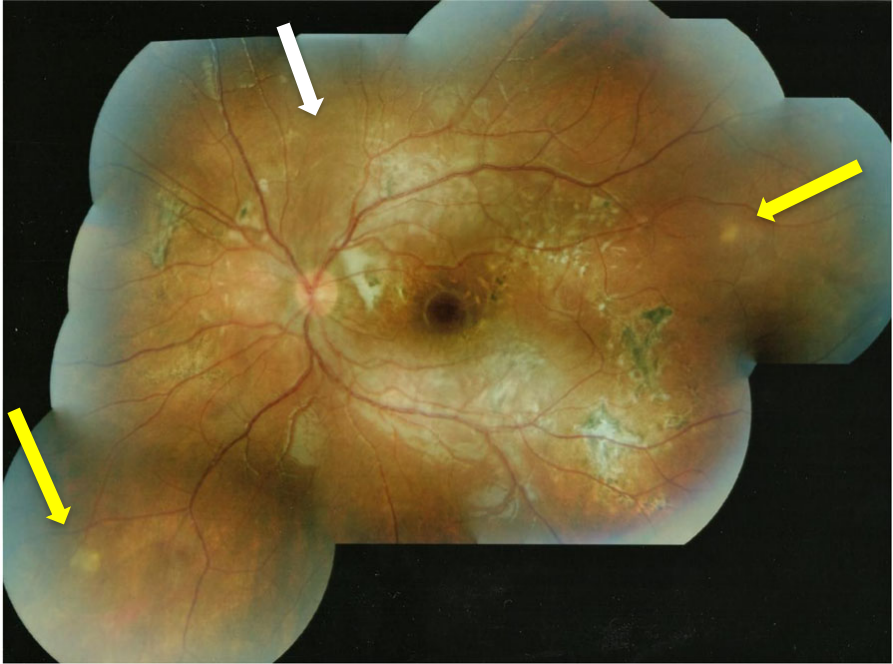
Colour fundal photograph of left eye 6 months following presentation demonstrating new active areas of chorioretinitis temporal to the superotemporal arcade and in the inferonasal retina (yellow arrows). Note the treated area superior to the optic disc had disappeared (white arrow)

**Fig. 6:**
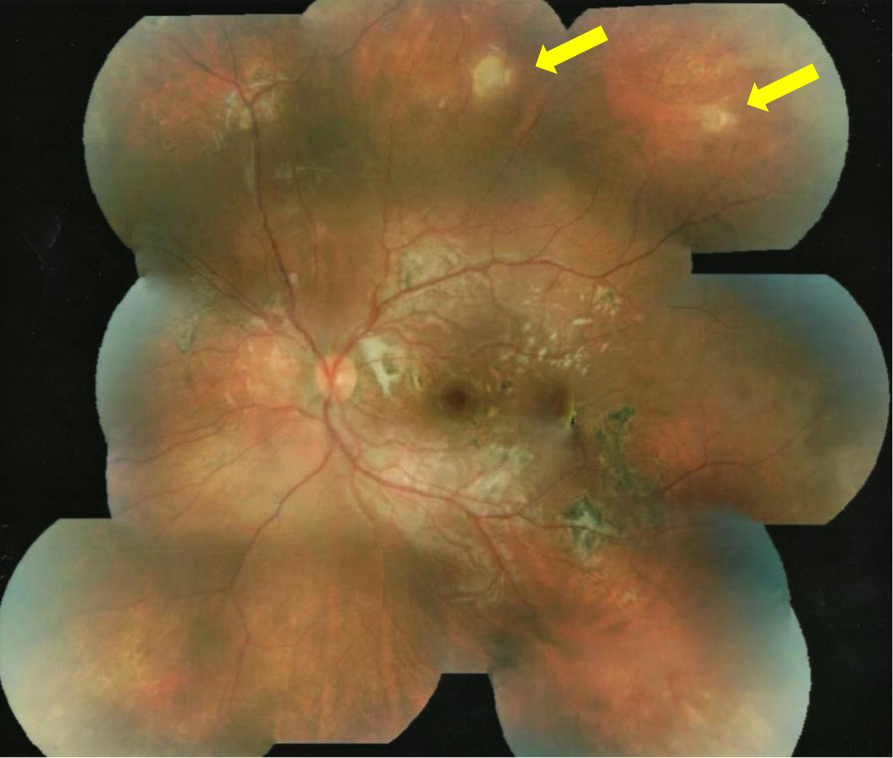
Colour fundal photograph of left eye 8 months following presentation demonstrating new areas of chorioretinitis in the superotemporal retina (yellow arrows) with resolution of areas inferonasally and temporal to the superotemporal arcade

Following diagnosis of DUSN, diode laser was performed under general anaesthetic to the superior area of active chorioretinitis and presumed nematode. The patient was also commenced on oral albendazole 200 mg twice daily for 1 month and a reducing course of oral prednisolone starting at 25 mg daily and tapered over 30 days. After another month, her visual acuity had improved further to 6/9 and the treated area of chorioretinitis superior to the optic disc had disappeared. However, new areas of chorioretinitis had appeared temporal to the superotemporal arcade and in the inferonasal fundus (Fig. 5). A further course of oral albendazole 200 mg twice daily and oral prednisolone 7.5 mg was commenced and planned for 30 days but was discontinued by the patient after 2 weeks. After a further 2 months, the fundal appearance had changed once again and new areas of chorioretinitis had appeared in the superotemporal retina with resolution of the areas inferonasally and temporal to the superotemporal arcade (Fig. 6). Further laser was administered to the new superior lesion where a nematode was suspected. Despite additional anti-helminthic treatment with ivermectin, recurrence of active chorioretinitis lesions continued and electrophysiology testing indicated significant left retinal dysfunction.

**Fig. 7:**
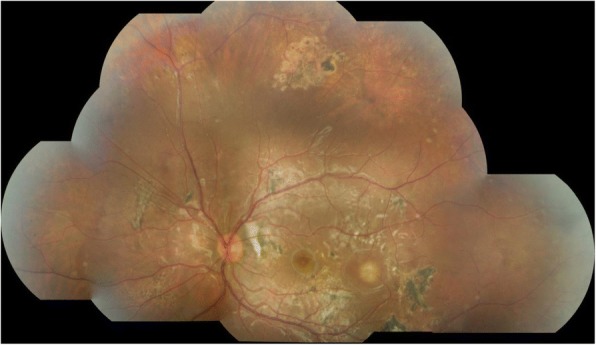
Colour fundal photograph of left eye 19 months following presentation demonstrating a new area of chorioretinitis at the temporal macula

A specialist uveitis opinion was sought from a tertiary centre in London that concurred with the diagnosis of DUSN; but it was thought that a secondary immune-mediated inflammatory response might be contributing to the clinical picture. Another tapering course of oral prednisolone along with mycophenolate mofetil was trialled without success. At last review, 19 months from initial presentation, the fundal appearance continued to change and a new area of focal chorioretinitis had appeared at the temporal macula (Fig. 7).

## Discussion

Diffuse unilateral subacute neuroretinitis (DUSN) is a rare cause of posterior uveitis in the United Kingdom (UK) [1]. Gass first described the condition in 1977 but the cause of inflammation remained unknown until association with a nematode was proposed in 1983 [2, 3]. It typically presents unilaterally in children and young adults but rarely bilateral cases have been reported [4].

Entry of the worm into the eye occurs when nematode eggs, after being ingested, find their way into the eye haematogenously. For months to years the worm can wander in the sub-retinal space [2]. The causative nematodes are of two types based on their length. Small nematodes (400-1000um) such as *Ancylostoma caninum* are endemic in South-eastern United States of America (USA), the Caribbean, Venezuela, Brazil and northern parts of South America [8]. Larger nematodes (1000-2000um), as suspected in this case, such as *Baylisacaris procyonis (B. procyonis)* are found in North and Midwestern USA, Europe and Brazil. *B. procyonis* is found abundantly in its definitive host, the racoon; hence DUSN is a disease rarely encountered in the UK [9]. However, the parasite has been found to have the ability to infect more than 90 kinds of wild and domestic animals. Many of these animals act as intermediary hosts and the infection results in the penetration of the gut wall by the larvae and subsequent invasion of tissue, resulting in disease. A number of reports have documented infection of domestic dogs with egg-laying adult *B. procyonis* worms. Because of their close contact with humans, particularly children, B. procyonis infection of domestic dogs represents a greater potential risk of infection and is a worrisome development.

Clinical features of DUSN can be divided into early and late. Early features include mild-to-moderate vitritis, papillitis, retinal vasculitis, grey–white or yellow–white evanescent lesions located in the deep retinal layers and retinal pigmentary epithelial (RPE) changes [5]. It is thought the active gray-white chorioretinal spots are an immune response to a secretion or excretion from the worm which often disappear in 1–2 weeks as the nematode moves elsewhere in the eye but pigment epithelial changes are also often seen indicating its travel pattern [10, 11]. Late features include multifocal choroiditis episodes, increase in the internal limiting membrane reflex (Oréfice’s sign), diffuse retinal atrophy, narrowing of retinal vessels and optic disc atrophy [5]. Our case demonstrated features of both early and late disease without the features of retinal arteriole narrowing and optic atrophy described in “unilateral wipe-out syndrome” [2].

This case highlights the difficulties often encountered in the treatment of DUSN, even when a worm can be identified. Laser photocoagulation remains the most successful treatment when the worm can be visualised; it is important to perform the laser as soon as the nematode is identified as it can quickly migrate out of sight. However, a worm can only be identified in 25–40% of patients [5, 6]. Treatment with oral albendazole is an option that has been used with variable success and a regime of 400 mg for 30 days is suggested [6]. However, our patient continued to develop new chorioretinal lesions despite treatment with 2 different anti-helminthic agents. Treatment of the disease in its early stages can arrest progression and prevent significant visual morbidity. Whilst the patient in this case retained visual acuity of 6/9 at more than 12 months following presentation, electrophysiology testing indicated significant left retinal dysfunction. We remain concerned for her long-term visual prognosis due to progression of clinical findings despite multiple attempts at laser photocoagulation and systemic anti-helminthic and immunosuppressive treatment. Although rarely reported [12], we have to consider the possibility of multiple worms in the sub-retinal space causing the clinical picture and frequent and careful examination is essential to identify this possibility. There is also a concern as to other central nervous system (CNS) involvement given her significant and ongoing headaches. However, evaluation by a paediatric infectious disease specialist has revealed no other CNS involvement through neuro-imaging or cerebrospinal fluid (CSF) analysis but repeating her MRI of brain in the future may be of value in demonstrating progression of the infection [13].

## Conclusion

We present a challenging case of DUSN in a young girl; a condition that remains rare in the UK. She was unresponsive to both focal laser and systemic anti-helminthic and immunosuppressive treatments suggesting the possibility of multiple worms being present in the sub-retinal space. Her visual prognosis is poor as there is ongoing recurrence of active chorioretinitis.

## Abbreviations

B. procyonis: Baylisacaris procyonis
CNS: Central nervous system
CRP: C-Reactive protein
CSF: Cerebrospinal fluid
DUSN: Diffuse unilateral subacute neuroretinitis
ESR: Erythrocyte sedimentation rate
MRI: Magnetic resonance imaging
PCR: Polymerase chain reaction
RPE: Retinal pigmented epithelium
UK: United Kingdom
USA: United States of America
